# First ICAN SSD survey- what do we have in Africa?

**DOI:** 10.1186/2047-2994-4-S1-P49

**Published:** 2015-06-16

**Authors:** B Hakizimana, S Mehtar

**Affiliations:** 1 Infection Prevention and Control, University Teaching Hospital of Butare, Huye, Rwanda; 2 Academic Unif for Infection Prevention and Control, Stellenbosch University, Cape Town, South Africa

## Introduction

Adequate reprocessing of reusable medical devices is vital to protecting patient safety. Therefore, inadequate or improper cleaning of reusable medical devices puts patients at risk for healthcare-associated infections (HAIs). There are few studies carried out in the field of decontamination and sterilization of reusable medical devices in Africa, thus the current situation of sterile services in Africa remains unknown.

## Objectives

This survey will help to establish needs in Africa for sterile services and education programmes.

## Methods

This survey was conducted during March and September 2014. The questionnaire developed and emailed to all ICAN Board members and PDIC students. The questionnaire composed by 6 components: hospital general information, CSSD design, reprocessing equipment, autoclaves, testing sterilizers, Sterile storage and CSSD environmental cleaning. 53 completed forms from 11 countries returned to the investigator. Data entered into access database, and then analysed with MS Excel.

## Results

62% of CSSD is managed by Nurse, and only 17% managed by CSSD qualified manager. 68% of CSSD fall under operating theatre. Only 4% of CSSD staff are SSD qualified. 38% of surgical devices cleaned only in CSSD. 60% surgical devices are cleaned manually 47% 62% sterilizers reported as good functioning. 43% perform Leak test daily, 56% Bowie Dick test, 60% chemical indicator is placed in each pack daily, 90% autoclave tape is placed on each pack daily, 60% biological indicator is performed daily.

## Conclusion

Ineffective surgical devices reprocessing, inappropriate CSSD design, lack of adequate training for CSSD staff, lack of appropriate and sufficient equipment, materials and supplies are the major obstacles to the effective CSSD service delivery in Africa. Adequate training of SSD staff should be taken as the first priority and provision of necessary equipment, supplies to be considered for better service delivery.

## Disclosure of interest

None declared.

